# Thoracic spinal anesthesia with intrathecal sedation for lower back surgery: a retrospective cohort study

**DOI:** 10.3389/fmed.2024.1387935

**Published:** 2024-04-11

**Authors:** Nikolay Boykov, Dilyan Ferdinandov, Petra Vasileva, Dimo Yankov, Stefan Burev, Rositsa Tanova

**Affiliations:** ^1^Department of Anesthesiology and Intensive Care, St. Ivan Rilski University Hospital, Sofia, Bulgaria; ^2^Clinic of Neurosurgery, St. Ivan Rilski University Hospital, Sofia, Bulgaria; ^3^Department of Neurosurgery, Faculty of Medicine, Medical University of Sofia, Sofia, Bulgaria; ^4^Department of Anesthesiology and Intensive Care, Faculty of Medicine, Medical University of Sofia, Sofia, Bulgaria

**Keywords:** thoracic spinal anesthesia, intrathecal midazolam, intrathecal clonidine, intrathecal dexmedetomidine, intrathecal sedation, spine surgery

## Abstract

**Background:**

Spinal anesthesia (SA) is a good alternative to general anesthesia (GA) for spine surgery. Despite that, a few case series concern the use of thoracic spinal anesthesia for short-duration surgical interventions. In search of an alternative approach to GA and a better opioid-free modality, we aimed to investigate the safety, feasibility, and patient satisfaction of thoracic SA for spine surgery.

**Materials and methods:**

We analyzed retrospectively a cohort of 24 patients operated on for a degenerative and osteoporotic pathology of the lower thoracic and lumbar spine. Data was collected from medical records, including clinical notes, operative and anesthesia records, and patient questionnaires.

**Results:**

Twenty-one surgeries for herniated discs, two for degenerative spinal stenosis, and one for multi-level osteoporotic vertebral body fractures were performed under spinal anesthesia with intrathecal sedation. In all cases, we applied 0.5% isobaric bupivacaine and the following adjuvants: midazolam, clonidine or dexmedetomidine, and dexamethasone. We boosted the anesthesia with local ropivacaine due to inefficient sensory block in two patients. Nobody in the cohort received intravenous opioids, non-steroidal anti-inflammatory drugs, or additional sedation intraoperatively. Postoperative painkillers were upon the patient’s request. No significant complications were detected.

**Conclusion:**

Thoracic spinal anesthesia incorporating adjuvants such as midazolam, clonidine or dexmedetomidine, and dexamethasone demonstrates not only efficient conditions for spine surgery, a favorable safety profile, high patient satisfaction, and intrathecal sedation but also effective opioid-free pain management.

## Introduction

Numerous studies have confirmed that spinal anesthesia (SA) is a good alternative to general (GA) for lower spine surgeries. It demonstrates a low level of intra- and postoperative complications, including cognitive impact in at-risk patients, and better postoperative pain management with reduced anti-inflammatory drugs and opioid utilization. Additionally, the SA is associated with decreased operative duration, time to ambulation, length of hospitalization, and costs compared to GA (1–5).

Anesthetic procedures at the thoracic and upper lumbar segment are far less common but are expected to offer similar advantages. The literature concerning the use of thoracic spinal anesthesia with intrathecal sedation for lumbar spine surgery is scarce. Only a few case reports and series with limited subjects have recently been published, and a widely accepted protocol is missing (6–8). Some clinicians have voiced concern about an increased risk of neurological deficits from injuring the spinal cord and difficulty in getting intrathecal access to perform spinal anesthesia in patients with degenerative vertebral pathology, especially with segmental vertebral deformities. However, some authors present results without an increased rate of complications (8, 9).

The aim of this study is to evaluate the feasibility, safety, patient satisfaction, and opioid-sparing potential of thoracic spinal anesthesia with intrathecal sedation for spine surgery.

## Materials and methods

All procedures discussed in this retrospective cohort study were conducted between March 2022 and December 2023 in the Clinic of Neurosurgery at St. Ivan Rilski University Hospital, Sofia, Bulgaria, a tertiary care facility for spinal and neurosurgical intervention. This work fulfills the STROBE checklist for reporting cohort observational studies. We analyzed a cohort of 24 patients operated on for a degenerative and osteoporotic pathology of the lower thoracic and lumbar spine.

Briefly, all patients received spinal anesthesia through a routine single-shot technique with a 22G Quincke needle in a sitting position. After identifying the intervertebral space by anatomical landmarks, 2 cm of the spinal needle was inserted by a paramedian approach. Any further insertion was performed with caution until bony contact with vertebral lamina. The spinal needle was then redirected and further advanced by 2–3 mm increments. After each advancement a check for cerebral spinal fluid backflow was performed. Once the needle was in the intrathecal space, 0.5% isobaric bupivacaine solution was applied in the range of 10–15 mg. Adjuvants, including an α-2 agonist (clonidine 10–20 mcg or dexmedetomidine 10–15 mcg), midazolam (2–3 mg), and dexamethasone (4 mg), were administered. Patients were then placed supine till the sensory block fixation and then in lateral decubitus or prone position for surgery. The level of puncture was verified by C-arm. No urethral catheters were inserted.

Postoperative pain management consisted of non-steroidal anti-inflammatory drugs (NSAID) on demand, including 1 g of paracetamol or 50 mg of dexketoprofen. Opioids were given if sufficient analgesia wasn’t achieved with the previous.

We used the Ramsay Sedation Scale (RSS) as a tool to evaluate the intraoperative level of consciousness, Table 1 (10). Pain intensity was assessed by the Visual Analogue Scale (VAS) presented by a straight line with points ranging from 0 (“no pain at all”) to 10 (“the worst possible pain”). It was measured at the 6th and 24th hour after the puncture for SA. Information about the level of patient satisfaction was retrieved from specific questionnaires designed by our group and given to all patients who underwent surgery under loco- regional anesthesia on the day of hospital discharge. The questionnaires included 3 questions, each with three possible answers, Table 2. Every patient with a sum of fewer than 7 points was considered satisfied, whereas we accepted a result of 7 as borderline.

**Table 1:** Ramsay sedation scale to assess patient’s consciousness level.

Clinical score	Patient characteristics
1	Awake, agitated or restless or both
2	Awake, cooperative, oriented, and tranquil
3	Awake, responds to commands only
4	Asleep, brisk response to light glabellar tap or loud auditory stimulus
5	Asleep, sluggish response to light glabellar tap or loud auditory stimulus
6	Asleep, no response to glabellar tap or loud auditory stimulus

**Table 2:** Patient satisfaction questionnaire, designed by our group, consists of 3 questions with three answers each.

Question to patients	Patient’s responses	Points
How did you feel during the anesthesia administration?	Totally relaxed	1
	Uneasy, concerned	3
	Anxious, stressed, scared	6
How would you rate your experience during the surgery?	Very pleasant	1
	Neither pleasant nor unpleasant	3
	Totally unpleasant	6
Would you choose the same anesthetic modality for a supposed surgery in the future (if applicable)?	I would surely choose it	1
	I cannot decide	3
	Most definitely not	6

Procedural time, puncture level, drug amounts, sensory blockade and sedation levels, patient and surgeon satisfaction, and postoperative usage of painkillers were analyzed for each case. Data was collected from medical records, including clinical notes, operative and anesthesia records and questionnaires.

## Results

The study cohort included 24 patients (11 females and 13 males) with a mean age of 49.6 years (range 21–88 years). All patients were grade I or II according to the physical status classification system of the American Society of Anesthesiologists (ASA). They suffered from disc herniations at the lumbar level, except two with degenerative spinal stenosis and one with multi-level osteoporotic compression vertebral fractures at the thoracolumbar junction. Patients’ data and details regarding the surgical intervention, spinal anesthesia, and early clinical outcome are extensively presented in Table 3.

**Table 3:** Patients’ data and details regarding the surgical intervention, spinal anesthesia, and early clinical outcome are.

ID	Age, sex	Diagnosis	Operative procedure	Surgical position	OR time	Surgery duration	Puncture level	Anesthesia level	Medication	VAS 24 h
1.	71 m	Degenerative spinal stenosis L4-L5 with polyradiculopathy	Hemilaminectomy L4 (left), foraminotomy L4-L5 (left) and over-the-top decompression	prone	115	50	L2-L3	T12	BUPI 15 mg, MDZ 3 mg, DEX 10 mcg	3
2.	64 m	HD L5-S1 with radiculopathy L5 (left)	Interlaminar approach L5-S1 (right), sequestrectomy and discectomy	prone	85	60	L1-L2	T2-T3	BUPI 15 mg, MDZ 2 mg	4
3.	45f	HD L5-S1 with radiculopathy S1 (right)	Interlaminar approach L5-S1 (right), sequestrectomy and discectomy	lateral decubitus	80	35	T12-L1	T7-T8	BUPI 15 mg, MDZ 2.5 mg, CLON 10 mcg	2
4.	36 m	HD L5-S1 with radiculopathy S1 (right) - recidive	Interlaminar approach L5-S1 (right), sequestrectomy and discectomy	prone	110	60	L2-L3	T10	BUPI 15 mg, MDZ 2 mg, CLON 15 mcg	5
5.	36f	HD L4-L5 with radiculopathy L4 and L5 (left)	Interlaminar approach L4-L5 (left), sequestrectomy and discectomy	prone	140	70	L3-L4	T12	BUPI 15 mg, ROPI 7.5 mg, MDZ 3 mg, CLON 20 mcg	3
6.	39f	HD L5-S1 with radiculopathy S1 (right)	Interlaminar approach L5-S1 (right), sequestrectomy and discectomy	prone	90	60	T12-L1	T1-T2	BUPI 15 mg, MDZ 3 mg, CLON 20 mcg	4
7.	72f	Osteoporotic compression fractures of T11, T12 and L1	Percutaneous transpedicular vertebroplasty T11, T12 and L1	prone	65	30	L1-L2	T11	BUPI 12.5 mg, MDZ 2.5 mg, CLON 20 mcg	4
8.	48 m	HD L5-S1 with radiculopathy S1 (right)	Interlaminar approach L5-S1 (right), sequestrectomy and discectomy	prone	65	30	T12-L1	T5	BUPI 15 mg, MDZ 2.5 mg, CLON 15 mcg	4
9.	35 m	HD L5-S1 with radiculopathy S1 (right)	Interlaminar approach L5-S1 (right), sequestrectomy and discectomy	prone	70	45	T12-L1	T2	BUPI 12.5 mg, MDZ 3 mg, CLON 20 mcg	3
10.	38 m	HD L5-S1 with radiculopathy S1 (right) - recidive	Interlaminar approach L5-S1 (right), sequestrectomy and discectomy	lateral decubitus	100	70	T12-L1	T8	BUPI 12.5 mg, MDZ 3 mg, CLON 20 mcg	1
11.	52f	HD L5-S1 with radiculopathy S1 (right)	Interlaminar approach L5-S1 (right), sequestrectomy and discectomy	prone	70	30	T12-L1	T7	BUPI 10 mg, MDZ 3 mg, CLON 20 mcg	2
12.	69f	HD L3-L4 with radiculopathy L4 (right) / Intradural sequester	Interlaminar approach L3-L4 (right), sequestrectomy, discectomy and dural repair	prone	165	120	T12-L1	T4	BUPI 12.5 mg, MDZ 3 mg, CLON 20 mcg	1
13.	44f	HD L4-L5 with radiculopathy L4 and L5 (left)	Hemilaminectomy L4 (left), foraminotomy L4-L5 (left), sequestrectomy and discectomy	prone	100	55	T12-L1	T5	BUPI 12.5 mg, MDZ 3 mg, CLON 20 mcg	2
14.	48 m	HD L5-S1 with radiculopathy S1 (left)	Interlaminar approach L5-S1 (left), sequestrectomy and discectomy	prone	90	40	T12-L1	T5	BUPI 12.5 mg, MDZ 3 mg, CLON 20 mcg	2
15.	88 m	Degenerative spinal stenosis L3-L4 with polyradiculopathy	Laminectomy L3 and partial laminectomy L4	prone	95	60	T12-L1	T5	BUPI 15 mg, MDZ 3 mg, DEX 15 mcg	4
16.	42 m	HD L5-S1 with radiculopathy S1 (left)	Interlaminar approach L5-S1 (left), sequestrectomy and discectomy	prone	60	30	T12-L1	T5	BUPI 12.5 mg, MDZ 3 mg, CLON 20 mcg	4
17.	38f	HD L5-S1 with radiculopathy S1 (right) - recidive	Interlaminar approach L5-S1 (right), sequestrectomy and discectomy	prone	75	40	T12-L1	T3	BUPI 10 mg, MDZ 3 mg, CLON 20 mcg	2
18.	60 m	HD L4-L5 with radiculopathy L5 (left)	Interlaminar approach L4-L5 (left), sequestrectomy and discectomy	prone	75	45	T11-T12	T5	BUPI 10 mg, MDZ 3 mg, CLON 20 mcg	5
19.	44 m	HD L2-L3 with radiculopathy L2 (left)	Interlaminar approach L2-L3 (left), foraminotomy and sequestrectomy	prone	100	80	T12-L1	T5	BUPI 12.5 mg, MDZ 3 mg, CLON 20 mcg	1
20.	48f	HD L4-L5 with radiculopathy L5 (left) and synovial cyst	Interlaminar approach L4-L5 (left), cystectomy, sequestrectomy and discectomy	prone	120	90	T12-L1	T5	BUPI 12.5 mg, MDZ 3 mg, CLON 20 mcg	3
21.	45f	HD L5-S1 with radiculopathy S1 (right) - recidive	Interlaminar approach L5-S1 (right), sequestrectomy and discectomy	prone	110	85	L1-L2	T10	BUPI 10 mg, MDZ 3 mg, CLON 20 mcg	3
22.	39 m	HD L3-L4 with radiculopathy L3 (left)	Translaminar approach L3-L4 (left) and sequestrectomy	prone	90	50	T11-T12	L1	BUPI 10 mg, ROPI 7.5 mg, MDZ 3 mg, CLON 20 mcg	3
23.	21 m	HD L4-L5 with radiculopathy L5 (right)	Interlaminar approach L4-L5 (right) and sequestrectomy	prone	75	60	T12-L1	T2-T3	BUPI 12.5 mg, MDZ 3 mg, CLON 20 mcg	3
24.	54f	HD L4-L5 with radiculopathy L5 (right)	Interlaminar approach L4-L5 (right), sequestrectomy and discectomy	lateral decubitus	65	45	T11-T12	T5	BUPI 12.5 mg, MDZ 3 mg, CLON 20 mcg	2

BUPI, isobaric bupivacaine 0.5%; ROPI, isobaric ropivacaine 0.5%; MDZ, midazolam; CLON, clonidine; DEX, dexmedetomidine; m, male; f, female. Dexamethasone 4 mg is applied in all cases.

The mean time spent by the patient in the operating room was 92 min (range 60–165 min, SD ± 26 min), and the surgical duration was 56 min (range 30–120 min, SD ± 22 min). In six cases, the point of access was at or below the L1-L2 level, whereas all the remaining dural punctures were in the T11-L1 segment, with the T12-L1 level being the most common in 15 procedures. The drug amounts were adjusted individually based on the puncture level, patient demographics, and comorbidities. We applied up to 12.5 mg of bupivacaine above L1-L2 with a single-shot technique and 15 mg at lower access points. Two patients (ID No 5 and 22) required an additional local application of 7.5 mg of ropivacaine due to an inefficient sensory block. In all cases, the spinal anesthesia was successful. Sedation, lasting approximately 45 min, was achieved at levels between 2 and 3 according to the Ramsay Sedation Scale in all cases. Nobody in the cohort received opioids, NSAIDs, or additional intravenous sedation intraoperatively.

Hemodynamic stability was maintained throughout the whole period of anesthesia, with a mean drop of the systolic blood pressure of 28 mmHg (range 10–50 mmHg, SD ± 19 mmHg). The mean drop of mean arterial pressure (MAP) was 15 mmHg (range 0–43 mmHg, SD ± 14 mmHg), corresponding to 18.1% (range 0–38.2%, SD ± 18.3%). One patient (ID No: 15, 88 year-old, degenerative spinal stenosis with laminectomy) developed a drop of MAP of 38.2% which required the use of a vasopressor (10 mg ephedrine intravenously).

Four patients had 6, fifteen had 5, five had 3, and two had 7 points on patient satisfaction scores assessed by our proprietary questionnaire. Thus, the satisfaction rate was 91.7%. The rest were borderline. Twenty patients reported that they would choose the same anesthetic modality in the future, whereas four could not decide. All patients reported an overall positive experience in the operating room.

The median reported VAS score both at 6th post-puncture hour was 2 (range 1–3) and 24th hour was 3 (range 1–5). Twenty-one out of 24 patients reported the need for postoperative analgesia with an NSAID. In all of them it occurred in the morning of surgery and during movement. In none of the cases opioids were required. All patients were ambulated on the same day and were discharged on postoperative days between 1 and 3.

The surgical conditions evaluated by the operator were optimal in all performed interventions, further supporting the feasibility of this technique. No intraoperative liquorrhea related to the spinal anesthesia was evident. No transient or permanent neurologic deficit was registered after dissipation of the sensory blockade. One patient developed transient urinary retention and a globus vesicalis, which was resolved after the insertion of a urinary catheter. No major complications related to the anesthesia or surgery were observed.

## Discussion

It is believed that spinal anesthesia is unsuitable and even contraindicated for patients with pathology of the spine mainly because of the normal anatomy compromise and the unpredictability of the local anesthetic spread. In this article, we present a cohort of 24 patients who underwent spine surgery for degenerative disorders and osteoporotic fractures under SA. The anesthesia was successful in all cases without major surgical or procedural complications.

Nevertheless, spinal anesthesia has been used for vertebral surgery, and large numbers of patients were treated, but dural punctures were typically performed at the lumbar spine (4, 5, 11–13). On the one hand, as Saifuddin et al. noted, the location of conus medullaris in a large adult population was shown to range from the middle third of T12 to the upper third of L3, mean at the lower third of L1 (14), which is a zone of risk for any interventions. On the other hand, Duniec et al. reported that the concordance rate between clinical examination and using assessment of level identification for the lumbar puncture is 64% among patients undergoing spinal anesthesia for lower limb surgery (15). Because of the uncertain and insufficient coverage of the sensory blockade in the cranial direction for interventions at the lumbar spine, we adopted the lower thoracic dural puncture technique. Our data shows that the difference in only one level of puncture (L1-L2 compared to T12-L1) provides a significant increase (5 dermatome levels) of local anesthetic spread without increasing the risk of conus medullaris injury. Using our protocol as described, the somatosensory block consistently reached a level between T2 and T7 (mean at T5 dermatome) for access points at T12-L1 and above. The lower puncture sites achieved a level up to T10, which was insufficient for completely anesthetizing the skin in the upper border of the surgical incision. Thus, it mandates the need for supplemental local anesthetic skin infiltration by the surgeon. The observed sensory block patterns suggest that the spread of the anesthesia correlates with the level of puncture rather than the concentration and volume of the local anesthetic used.

The use of intrathecal sedation with midazolam and an α-2 agonist (either clonidine or dexmedetomidine) not only mitigated their hemodynamic and respiratory drive suppression effects, compared to when applied intravenously but also provided patient comfort during the procedure (7). This approach offers better hemodynamic stability than traditional SA without adjuvants with a lesser mean drop of MAP. The last provides an opportunity for its use in the elderly or comorbid patients. Vital signs are more stable than when emerging from GA and during the immediate postoperative period, which may be beneficial for patients with severe cardiac illness (16, 17). We confirm these findings with only one case at the age of 88 with a temporary and not clinically significant drop of MAP.

Importantly, none of the patients in our cohort required any additional sedation different from the described. Furthermore, no intra-procedural opioids were administered for pain management, indicating adequate analgesia without the need for traditional opioid-based approaches and even the use of NSAIDs. In our study, a good level of sedation lasted approximately 45 min. All patients reported an overall positive experience during surgery and an excellent satisfaction rate. This observation is supported by other authors using both benzodiazepines and dexmedetomidine (17).

Few articles present patient and surgeon satisfaction when comparing SA to GA (18, 19). We carefully prepared our patient satisfaction questionnaire to provide insight into the overall patient experience with the modality and compare pre- and postoperative patient comfort. The procedures were explained in great detail, and directions were given to all the patients. They were instructed to signal the anesthetist or the surgeon if any discomfort occurred because of stress, fear, pain, body position, etc. None of the patients had any of the mentioned complaints. To note, despite being lightly sedated, they responded well to commands and were cooperative overall. No involuntary movements were observed, which can create difficulties for the surgeon working under magnification.

In our study, all surgeries were performed by the same team. The operators were asked to evaluate the surgical conditions in terms of ease of obtaining the surgical field, patient positioning, operative room stay, and the feasibility of the intervention. In contrast with Sadrolsadat et al. (20) study, which showed SA had no advantages over GA, our surgical team evaluated the conditions as optimal. This confers with the findings of McLain et al. (21) with a focus on easier patient positioning, shorter operative room stay, and better facility management than with GA to further support the spinal anesthesia feasibility.

As many authors advocate, we also support the opioid-free options for anesthesia in spine surgery (22). No intrathecal or intravenous opioids were used in our cohort, and the postoperative painkillers were on demand. Patients were instructed to demand medications if pain level rises above VAS score 4 or discomfort is high. The staff was instructed to be vigilant about subjects requiring additional analgesia and/or complaining of insufficiency of analgesia by NSAIDs and the need for opioids. Out of the protocol, the patients were also asked at discharge to describe when and how the highest level of pain occurred, with the majority reporting pain at the surgical skin incision, only when moving, and in the following morning after the procedure, not exceeding VAS score 5. Adequate analgesia was achieved in all cases only with NSAIDs, while four patients did not need any painkillers.

Early ambulation was achieved in all 24 patients without any complications or neurologic deficits, which again highlights the safety and efficacy of thoracic spinal anesthesia with intrathecal sedation. We could not find any other study investigating these circumstances. In none of the patients, a urinary catheter was inserted before surgery, and fluid administration was cautious. Nevertheless, one patient (female, 34 years) developed a globus vesicalis, which was treated successfully, and no micturition disturbances were reported.

While our study provides valuable insights, certain limitations should be acknowledged. The relatively small sample size and the absence of a control group warrant caution in generalizing the results to broader patient populations. All patients being ASA I-II limits the findings to patients without severe comorbidities. However, it would be specifically appropriate for the high-risk groups. Therefore, further research is needed to explore the applicability and safety of thoracic spinal anesthesia in patients with more significant health challenges. Building on the positive outcomes observed in this study, future research should consider prospective trials with larger sample sizes to validate further the safety, efficacy, and cost-effectiveness of thoracic spinal anesthesia. Exploring the long-term effects, particularly concerning postoperative recovery and complications, would contribute to a more comprehensive understanding of its applicability in diverse clinical scenarios.

## Conclusion

Thoracic spinal anesthesia incorporating adjuvants such as midazolam, clonidine or dexmedetomidine, and dexamethasone demonstrates not only efficient conditions for spine surgery, a favorable safety profile, high patient satisfaction, and intrathecal sedation but also effective opioid-free pain management. Thus, our findings imply that this is an appropriate alternative to the general anesthesia for spine surgery. Future research should further investigate and validate the potential of the technique, including its cost-effectiveness, and explore the optimal surgical and pain management strategies.

## Data availability statement

The original contributions presented in the study are included in the article/supplementary material, further inquiries can be directed to the corresponding author.

## Ethics statement

Ethical approval was not required for the study involving humans in accordance with the local legislation and institutional requirements. Written informed consent to participate in this study was not required from the participants or the participants’ legal guardians/next of kin in accordance with the national legislation and the institutional requirements.

## Author contributions

NB: Data curation, Formal analysis, Investigation, Methodology, Writing – original draft. DF: Formal analysis, Project administration, Resources, Supervision, Visualization, Writing – review & editing. PV: Investigation, Project administration, Resources, Writing – original draft. DY: Formal analysis, Resources, Supervision, Writing – review & editing. SB: Investigation, Resources, Validation, Writing – original draft. RT: Conceptualization, Formal analysis, Investigation, Methodology, Project administration, Resources, Supervision, Writing – review & editing.
